# Implications of multimorbidity on healthcare utilisation and work productivity by socioeconomic groups: Cross-sectional analyses of Australia and Japan

**DOI:** 10.1371/journal.pone.0232281

**Published:** 2020-04-28

**Authors:** Grace Sum, Marie Ishida, Gerald Choon-Huat Koh, Ankur Singh, Brian Oldenburg, John Tayu Lee

**Affiliations:** 1 Saw Swee Hock School of Public Health, National University of Singapore, Singapore, Singapore; 2 Nossal Institute for Global Health, Melbourne School of Population and Global Health, University of Melbourne, Melbourne, Australia; 3 Centre for Health Equity, Melbourne School of Population and Global Health, University of Melbourne, Melbourne, Australia; 4 Department of Primary Care and Public Health, School of Public Health, Imperial College London, London, England, United Kingdom; Anglia Ruskin University, UNITED KINGDOM

## Abstract

**Background:**

Multimorbidity, the presence of 2 or more non-communicable diseases (NCDs), is a major contributor to inequalities of health in Australia and Japan. We use nationally representative data to examine (i) the relationships between multimorbidity with healthcare utilisation and productivity loss and (ii) whether these relationships differed by socioeconomic groups.

**Methods:**

Cross-sectional analyses using the Household, Income, and Labour Dynamics in Australia (HILDA) and the Japanese Study of Aging and Retirement (JSTAR) surveys. We examined 6,382 (HILDA) and 3,503 (JSTAR) adults aged ≥50 years. We applied multivariable regression, logistic and negative binomial models.

**Results:**

Prevalence of multimorbidity was overall 38.6% (46.0%, 36.1%, 28.9% amongst those in the lowest, middle and highest education group, respectively) in Australia, and 28.4% (33.9%, 24.6%, 16.6% amongst those in the lowest, middle and highest education group, respectively) in Japan. In Australia and Japan, more NCDs was associated with greater healthcare utilisation. In Australia and Japan, more NCDs was associated with higher mean number of sick leave days amongst the employed and lower odds of being employed despite being in the labour force. The association between multimorbidity and lower retirement age was found in Australia only.

**Conclusion:**

Having more NCDs pose significant economic burden to the health system and wider society in Australia and Japan. Targeted policies are critical to improve financial protection, especially for lower income groups who are more likely to have multiple NCDs. These individuals incur both high direct and indirect costs, which lead to a greater risk of impoverishment.

## Author summary

**What is known and unknown:**

- In Australia and Japan, it has been shown that more non-communicable diseases (NCDs) is associated with greater health service use.
- There is a lack of research in Australia and Japan on how more NCDs is associated with productivity loss, especially involuntary early retirement, absence from work due to chronic illness, and being unemployed despite being in the labour force.
- There are also limited studies on how the socioeconomic status of individuals in Australia and Japan influences the relationships between multimorbidity with healthcare utilisation and productivity loss.

**What this study adds:**

- In both Australia and Japan, having more NCDs is associated with higher healthcare utilisation and lower work productivity, including sickness absence, unemployment and early retirement.
- Across all levels of income and levels of education, there was an association between number of NCDs with healthcare utilisation and work productivity loss. Targeted policies are critical to improve financial protection, especially for lower education groups who are more likely to have multiple NCDs. These individuals incur both high direct and indirect costs, which lead to a greater risk of impoverishment

## Introduction

Non-communicable diseases (NCDs) are the leading cause of premature morbidity and mortality [1–4], and is a major contributor to health inequalities in many countries [5,6]. In Australia, about a third of the population have multimorbidity, with 17% of the population suffering from complex multimorbidity whereby 3 or more body systems are affected by at least 1 NCD [7]. In Japan, a study on elderly aged 75 years and above in Tokyo found that the prevalence of those with 3 or more NCDs was 65%, and multimorbidity was associated with increased number of outpatient visits and hospital admissions [8]. As the populations in Australia and Japan continue to face an exponentially ageing population and an increased exposure to risk factors [9,10]. multimorbidity will likely worsen.

Evidence from high-income countries has established that apart from negative health-related outcomes, multimorbidity imposes significant economic costs to individuals [11,12]. Studies have shown that patients with more NCDs have higher healthcare utilisation, such as having more outpatient visits, hospitalisations, medical equipment, and medicines [13]. These economic costs from higher treatment burden may not only include substantial medical expenditures, but also encompass loss of potential income due to involuntary absence from work [14–16]. There are only a few studies on the associations between multimorbidity and productivity in high-income countries, including Australia and Japan, and no study has used nationally representative datasets to the best of our knowledge.

The current literature has highly heterogenous findings on work productivity, and the existing studies have different populations, study designs, range of NCDs, and outcome measures [17–21]. Existing studies have also primarily examined productivity loss and decrease in work performance in working adults [14,22]. Importantly, studies have been primarily on the impact of self-perceived ill health or single NCDs like hypertension, diabetes, or mental illness [23,24], instead of multimorbidity, in terms of increasing numbers of NCDs. There is a need to fill the knowledge gap on how increasing number of NCDs is associated with work productivity loss in nationally representative populations. In addition, the burden of being unemployed and having to retire early, is likely compounded by the treatment burden of multimorbidity, such as higher healthcare utilisation. Also, majority of the previous studies have not investigated whether there is an association between number of NCDs with healthcare utilisation and work productivity loss, across all levels of income and levels of education [14,15,22,25,26].

This study utilises nationally representative data at the population level to examine (i) the relationships between multimorbidity with healthcare utilisation and productivity (ii) whether these associations differed by socioeconomic groups in both countries.

## Methods

### Sample and data

This study conducted secondary data analyses using cross-sectional data from the Household, Income, and Labour Dynamics in Australia survey (HILDA) Wave 17 (2017–2018), and the Japanese Study of Aging and Retirement survey (JSTAR) (2013).

The HILDA survey is a household-based panel study on nationally representative Australian residents aged 15 years and above. The HILDA survey is an annual survey that provides longitudinal data on personal well-being, economics, labour market dynamics and family life [27]. It was conducted to provide insights about Australia to policymakers in the areas of health, education and social services [27]. The JSTAR survey is a longitudinal study on nationally representative samples of subjects aged 50 years and above from Japanese residents of 10 cities across Japan [28]. The JSTAR survey was conducted by the Research Institute of Economy, Trade and Industry (RIETI), Hitotsubashi University, and the University of Tokyo, and covers in depth information regarding living aspects, including the economic, social, and health conditions of older adults [28].

Majority of current studies focus on the working population (i.e. employed at time of research study) and their decrease in work performance [14,22], with a dearth in studies on how having more NCDs impacts the involuntary exit from the labour force due to unemployment and early retirement. There are also currently little to no studies that use nationally representative datasets to examine multiple chronic diseases and work productivity loss. The HILDA survey Wave 17 and the JSTAR survey Wave 4 are one of the few high-quality surveys on nationally representative populations in HICs that included questions on the different aspects of work productivity like sickness absence, being unemployed while in the labour market, and early retirement, healthcare utilisation, as well as multiple NCDs [29–32].

For the HILDA survey, households were selected using a multi-staged approach. A stratified random sample of 488 Census Collection Districts, which contained 200 to 250 households in each district, was selected from across Australia [29,30]. A random sample of 22 to 34 dwellings were then selected based on expected response and occupancy rates of the areas, and within each dwelling, up to 3 households were randomly selected [29,30]. Smaller states and territories were not over-sampled to produce nationwide representative population estimates [29,30]. For data collection, there were approximately 175 interviewers in Wave 17, with 145 interviewers conducting face-to-face interviews and 30 interviewers conducting telephone interviews [29,30].

For the JSTAR survey, subjects were selected using multi-staged sampling, whereby pre-defined sites within each municipality were randomly selected, followed by randomly selecting individuals within each site [31,32]. The samples were also weighted based on socio-demographics like age, city of residence, and employment status. Collaborative efforts with governmental officials in each municipality allowed better response rates [31,32]. At the time of this study, the JSTAR survey had conducted 4 survey waves (2007, 2009, 2011, 2013), with the most recent wave in 2013 including data collection on both healthcare utilisation and labour force participation [31,32].

Both datasets were nationally representative as HILDA was weighted for the multi-stage sampling technique, and the sample from JSTAR was weighted based on socio-demographics like age, city of residence, and employment status.

The response rate for HILDA and JSTAR was 96.4% and 82.9%, respectively. For HILDA, our study included respondents aged 50 years and above (n = 7,285 of 23,415), and subsequently excluded respondents with missing data on covariates and NCDs (remaining n = 6,382 of 7,285; dropped 12.4%). JSTAR had 4,091 respondents aged 50 years and above, and we subsequently excluded the respondents with missing data on covariates and chronic conditions (remaining n = 3,503 of 4,091; dropped 14.4%). (S1 and S2 Figs). The amount of missing data was checked to be missing at random, and hence there was a low likelihood of selection bias. This means that the associations between exposures and outcomes were not different among those with missing data.

#### Ethics statement

This study obtained ethics approval from the National University of Singapore Institutional Review Board (NUS-IRB) with reference code S-19-178. In addition, all the data were fully anonymised before we accessed them, and participants provided written consent for their responses in the WHO SAGE survey to be used for research purposes.

### Variables

#### Predicting variable

The predicting variable was the number of NCDs each subject self-reported. Subjects were defined to have multimorbidity if they had two or more NCDs.

The HILDA survey had questions on 12 NCDs, which included arthritis/osteoporosis, asthma, cancer, chronic bronchitis/emphysema, type 1 diabetes, type 2 diabetes, depression, anxiety, other mental illness, heart disease, high blood pressure/hypertension, and any other serious circulatory condition. We defined respondents as having the NCD if they answered affirmatively to the following question: “Have you been told by a doctor or nurse that you have any of these conditions?”.

The JSTAR survey had questions on 18 NCDs, which included, the 18 NCDs included heart disease, high blood pressure, hyperlipidemia, cerebral /cerebrovascular accident, diabetes, chronic lung disease, asthma, liver disease, ulcer/other gastrointestinal disorder, joint disorder, osteoporosis, eye disease, ear disorder, bladder disorder, Parkinson’s disease, depression/emotional disorder, dementia, and cancer. We defined respondents as having the NCD if they answered affirmatively to any one of the following 3 questions: “Have you been newly diagnosed with or advised to seek medical advice for any of the listed illness since the time of the last interview?”, or “If you are receiving treatment for any other illness, is it the same illness as what you had at the time of the last interview?”, or “Is the illness relapse of an illness that you had prior to the last interview?”. These additional and relapsed illnesses still refer to the 18 NCDs in the survey.

#### Outcome variables

The 2 outcome variables were healthcare utilisation and work productivity loss. Healthcare utilisation referred to the mean number of outpatient visits in the past 12 months, and the mean number of nights spent in hospital in the past 12 months. Productivity loss was assessed via three outcomes: mean retirement age for retired subjects, mean number of days of sick leave in the past 12 months for respondents who are employed, and the odds of being unemployed despite being in the labour force. We defined individuals in the labour force as subjects who are employed as well as those who are unemployed but actively looking for employment. Individuals who are not in the labour force are commonly stay-at-home parents, retired, studying, and voluntarily unemployment. Detailed survey questions are in S1 Table.

All subjects were stratified by education level, to study the effect of educational attainment on the associations between multimorbidity with healthcare utilisation and productivity loss. For HILDA, education levels were: (i) lower education (year 11 and below) (ii) mid-level education (year 12, certificate 3 or 4, advanced diploma, diploma) (iii) higher education (bachelor or honours, graduate diploma, graduate certificate, post-graduate). For JSTAR, education levels were: (i) lower education (no education, primary school, middle school) (ii) mid-level education (high school, junior college) and (iii) higher education (university, technical college, graduate school).

#### Covariates

For HILDA, covariates were age (50–59, 60–69, 70–79, 80+ years), sex, education level (low, middle, high), employment status (employed, not employed), household income (Q1-Q4), marital status, residence (rural, urban), geographical state, country of birth, Aboriginal status, BMI (underweight, normal, overweight, obese), physical activity (low, middle, high), alcohol intake (no alcohol intake, low alcohol intake, high alcohol intake), and smoking (non-smoker, ex-smoker, current smoker).

For JSTAR, covariates were age (50–59, 60–69, 70–79, 80+), sex, education level (low, middle, high), employment status (employed, not employed), marital status, BMI (underweight, normal, obese), physical activity (little, moderate, heavy), alcohol intake (no alcohol intake, low alcohol intake, high alcohol intake), and smoking (non-smoker, ex-smoker, current smoker). Household income was not available in JSTAR.

#### Statistical analysis

The analyses were conducted on HILDA and JSTAR separately. It was not a comparative study. We summarised sample characteristics for each dataset. We presented the prevalence of individual NCDs, and the prevalence of multimorbidity unstratified and stratified by education level and age. Multivariable negative binominal, linear, and logistic regression models were used to examine associations between multimorbidity and each outcome (S2 Table). Specifically, the multivariable negative binomial regression model was fitted for the outcomes of mean number of outpatient visits, mean number of nights in a hospital, and mean number of sick leave days given the skewed nature of count data. The multivariable linear regression model was used to examine the continuous outcome of mean retirement age. Finally, multivariable logistic regression model was applied to examine unemployment despite being in the labour force (binary outcome).

We adjusted for covariates listed above. These sociodemographic covariates were considered important potential confounders for the relationships between number of NCDs with healthcare utilisation and work productivity. All the regression models were tested for collinearity.

To assess the impact of socioeconomic status on the associations between multimorbidity with healthcare utilisation and productivity loss, subjects were stratified by education level (both HILDA and JSTAR) and income quintiles (only HILDA, as JSTAR did not have data on household income), and the same analyses for healthcare utilisation and productivity loss (i.e. multivariable negative binominal, linear, and logistic regression models) were conducted. Income in the HILDA survey referred to actual earnings and not assets.

We performed the analyses using Stata 15 (Stata Corp.) and at 5% level of statistical significance.

## Results

### Sample characteristics

Prevalence of multimorbidity was overall 38.6% (46.0%, 36.1%, 28.9% amongst those in the lowest, middle and highest education group, respectively) in Australia, and overall 28.4% (33.9%, 24.6%, 16.6% amongst those in the lowest, middle and highest education group, respectively) in Japan. Table 1 shows the sample characteristics of subjects in HILDA and JSTAR separately. S3 and S4 Figs show the prevalence of individual NCDs.

**Table 1:** Sample characteristics.

	Australia (HILDA survey)	Japan (JSTAR survey)
	N = 6382	N = 3503
**Sex, N (%)**		
Male	3029 (49.5)	1710 (47.1)
Female	3353 (50.6)	1793 (52.9)
**Age, in years, N (%)**		
50–59	2438 (38.9)	530 (29.9)
60–69	2058 (32.0)	1505 (41.5)
70–79	1295 (19.9)	1335 (25.9)
80+	591 (9.2)	133 (2.8)
**Marital status, N (%)**		
Married	4453 (71.3)	2831 (93.7)
Not married	390 (5.9)	154 (2.3)
Others	1539 (22.9)	518 (4.0)
**No. of NCDs, N (%)**		
None	2023 (32)	1296 (37.0)
1	1894 (29.3)	1214 (34.7)
2	1254 (19.4)	577 (16.5)
3	688 (10.6)	254 (7.3)
4+	523 (8.6)	162 (4.6)
**Education level, N (%)**		
Low	2055 (32.2)	816 (22.8)
Middle	2755 (44.2)	2069 (57.6)
High	1572 (23.6)	618 (19.6)
**Annual household income, N (%)**		
Q1 (lowest)	1759 (31.6)	No data available
Q2	1065 (22.9)	
Q3	872 (19.5)	
Q4 (highest)	1097 (26.1)	
**Residence (urban versus rural), N (%)**		
Urban	5388 (86.0)	No data available
Rural	994 (14.0)	
**State, N (%)**		
New South Wales	1922 (31.9)	Not applicable
Victoria	1507 (24.8)	
Queensland	1339 (20.0)	
South Australia	615 (8.12)	
Western Australia	620 (10.5)	
Tasmania	223 (2.5)	
Northern Territory	39 (0.8)	
Australian Capital Territory	117 (1.4)	
**Aboriginal status, N (%)**		
Non-aboriginal	6307 (98.9)	Not applicable
Aboriginal/Tress Strait Island	75 (1.1)	

HILDA = Household, Income, and Labour Dynamics Australia; JSTAR = Japanese Study of Ageing and Retirement; NCDs = Non-communicable diseases.

**Fig 1:**
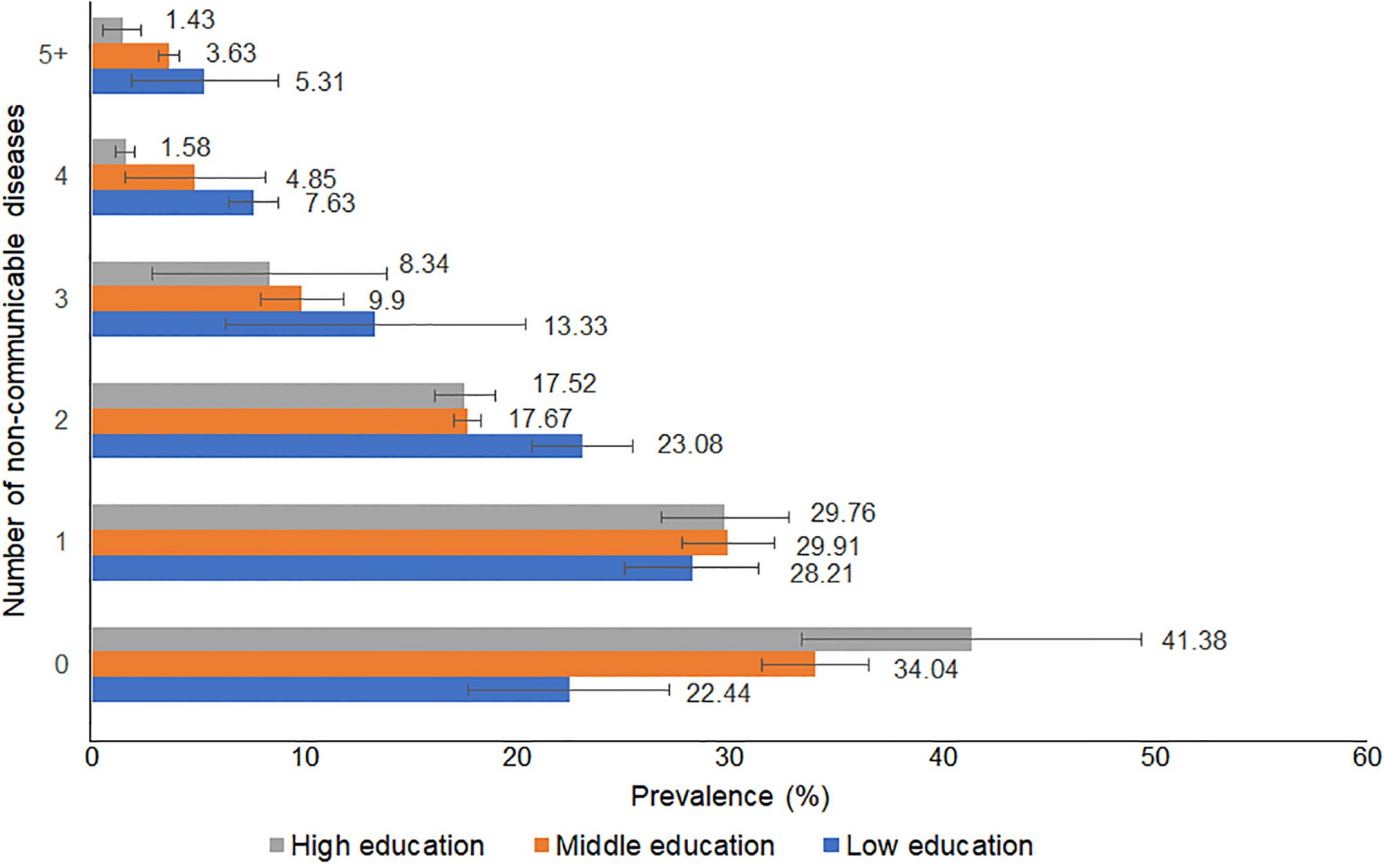
Prevalence of multimorbidity stratified by education level (Australia)^+^. ^+^The 12 non-communicable diseases (NCDs) include arthritis, cancer, type 1 diabetes, type 2 diabetes, heart diseases, hypertension, asthma, bronchitis, depression, anxiety, other mental diseases, circulatory conditions.

**Fig 2:**
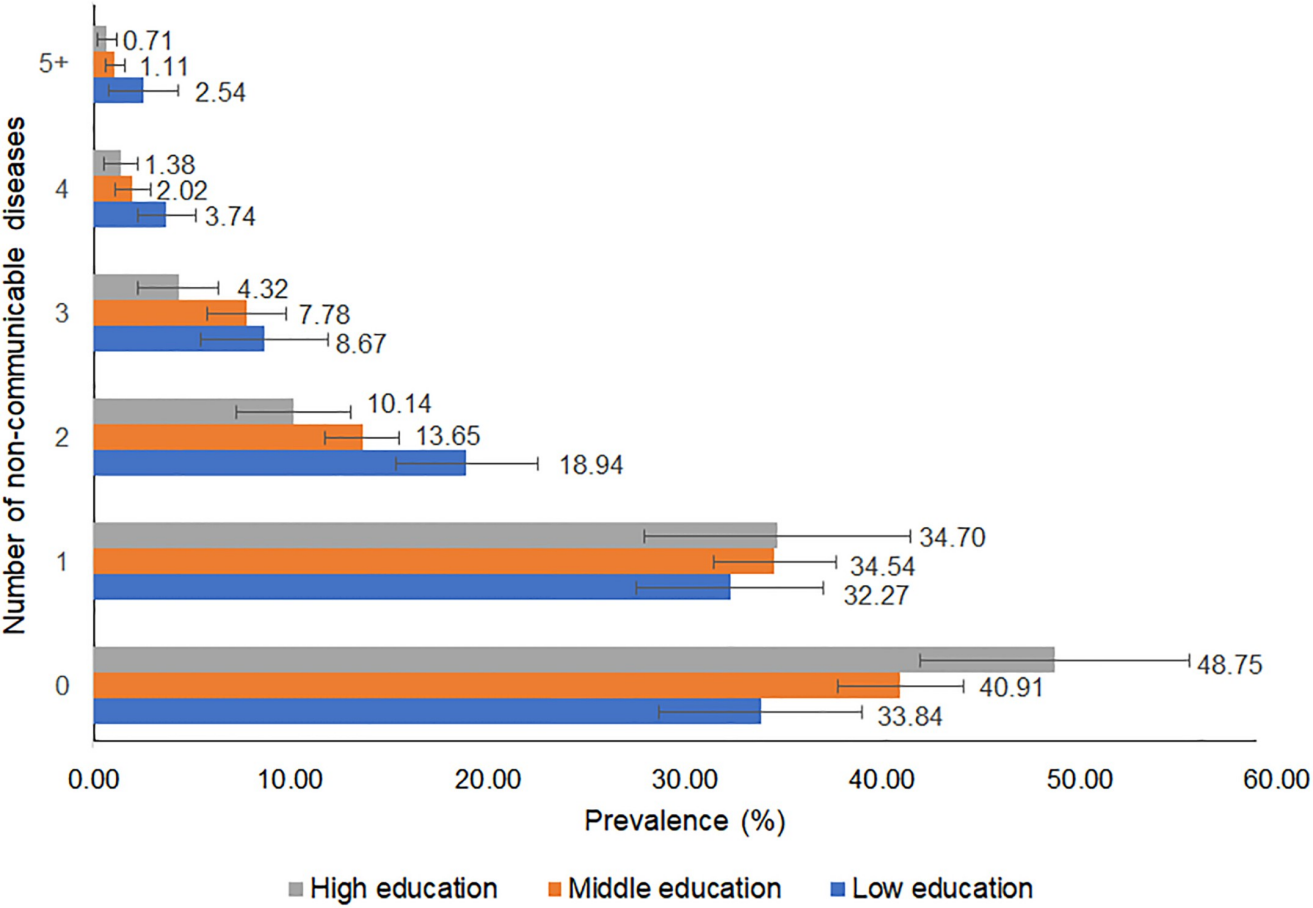
Prevalence of multimorbidity stratified by education level (Japan) ^+^. ^+^The 18 non-communicable diseases (NCDs) include heart disease, hypertension, cerebral/cerebrovascular accident, diabetes, chronic lung disease, asthma, joint disorder, depression or emotional disorder, cancer, hyperlipidaemia, liver disease, stomach ulcer or other stomach disorder, osteoporosis, eye disease, ear disorder, bladder disorder, Parkinson’s disease, dementia.

Figs 1 and 2 show the prevalence of multimorbidity (with errors bars of 95%CIs) stratified by education level in each country. In Australia and Japan, the prevalence of subjects with no NCDs was the greatest for those in the highest education group and lowest for those in the lowest education group. In contrast, the prevalence of subjects with 2 or more NCDs was greatest for those in the lowest education group and lowest for those in the highest education group.

**Fig 3:**
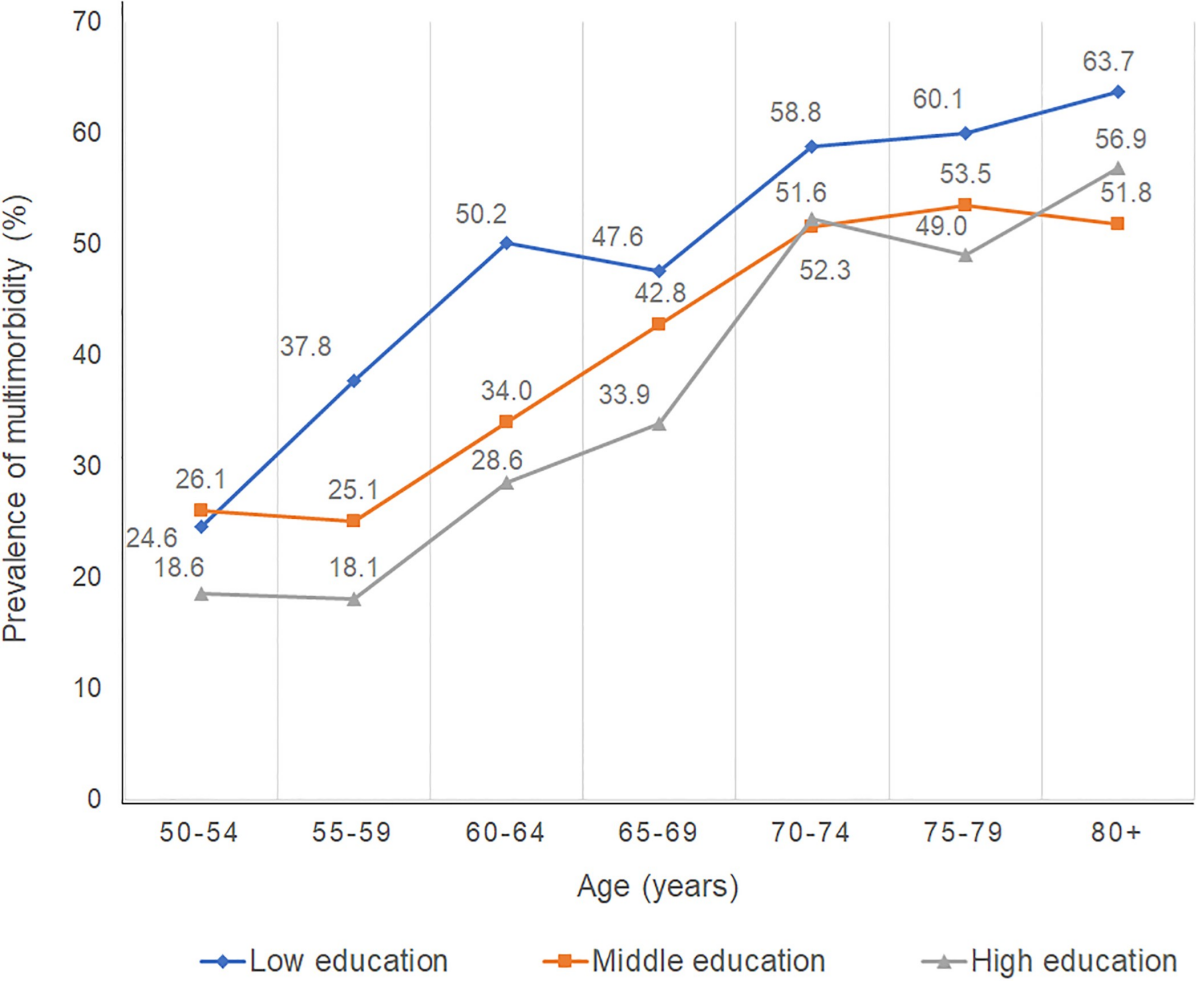
Prevalence of multimorbidity stratified by education level and age (Australia)^+^. ^+^The 12 non-communicable diseases (NCDs) include arthritis, cancer, type 1 diabetes, type 2 diabetes, heart diseases, hypertension, asthma, bronchitis, depression, anxiety, other mental diseases, circulatory conditions.

**Fig 4:**
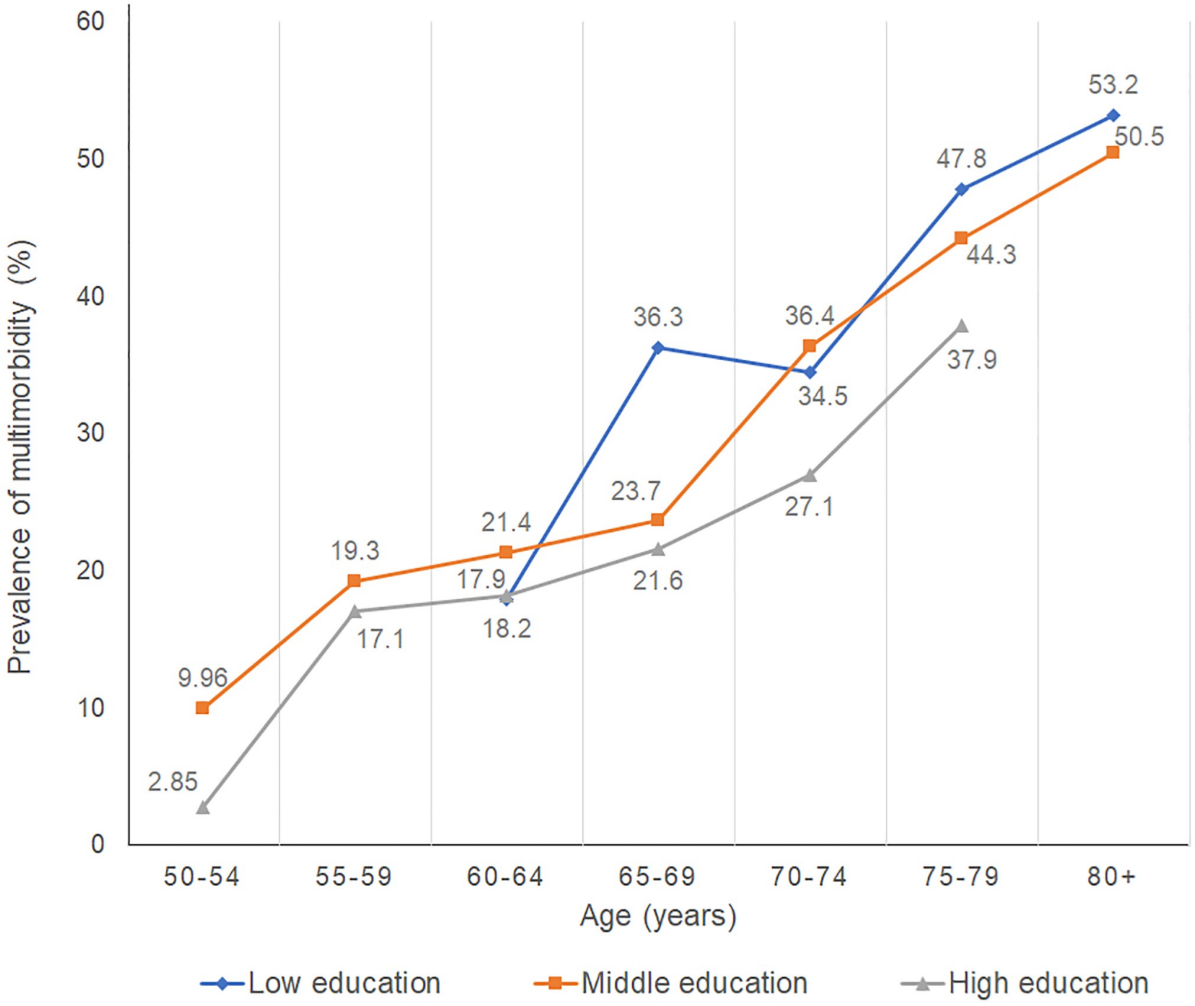
Prevalence of multimorbidity stratified by education level (Japan)^+^ (prevalence excluded if subgroup has <30 subjects). ^+^The 18 non-communicable diseases (NCDs) include heart disease, hypertension, cerebral/cerebrovascular accident, diabetes, chronic lung disease, asthma, joint disorder, depression or emotional disorder, cancer, hyperlipidaemia, liver disease, stomach ulcer or other stomach disorder, osteoporosis, eye disease, ear disorder, bladder disorder, Parkinson’s disease, dementia.

Figs 3 and 4 show the prevalence of multimorbidity stratified by both education level and age in each country. In Australia and Japan, the prevalence of multimorbidity increases as age increases; and in every age group, the prevalence of multimorbidity is highest for subjects in the lowest education group.

### Healthcare utilisation

#### Australia

**Fig 5:**
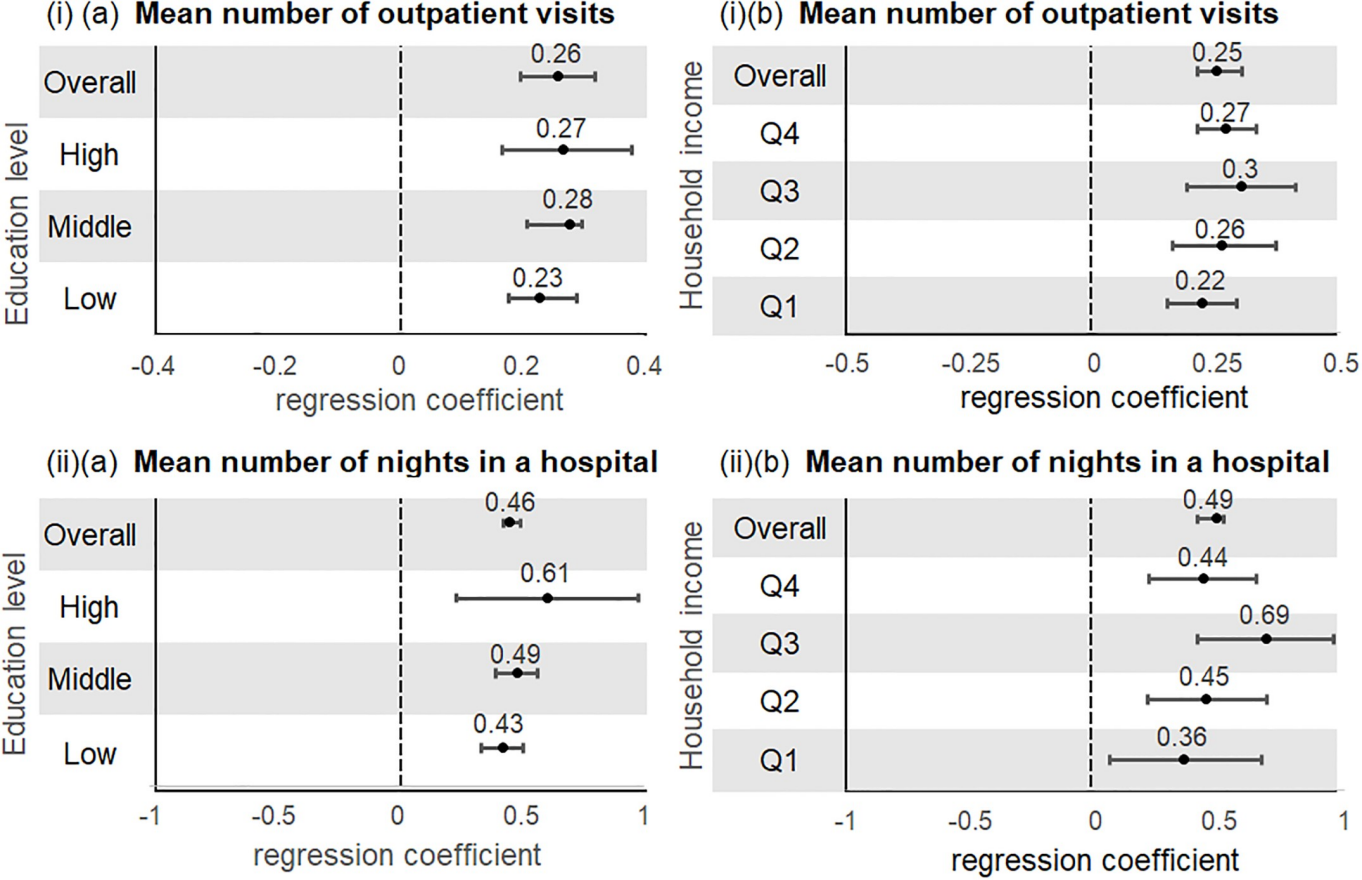
Associations between multimorbidity with mean number of (i) outpatient visits and (ii) nights in hospital by (a) education level and (b) household income (Australia)^+^. ^+^Multivariable negative binomial regression models were adjusted for age, sex, marital status, employment, household income (if stratified by education)/education (if stratified by household income), geographical status, country of birth, aboriginal status, body mass index, physical activity, alcohol intake, and smoking.

An increasing number of NCDs was associated with a higher mean number of outpatient visits and mean number of nights in a hospital (ß coefficients >0) (Fig 5). There were no statistically significant differences among socioeconomic groups. However, there appears to be a positive trend between number of nights in a hospital with income level.

#### Japan

**Fig 6:**
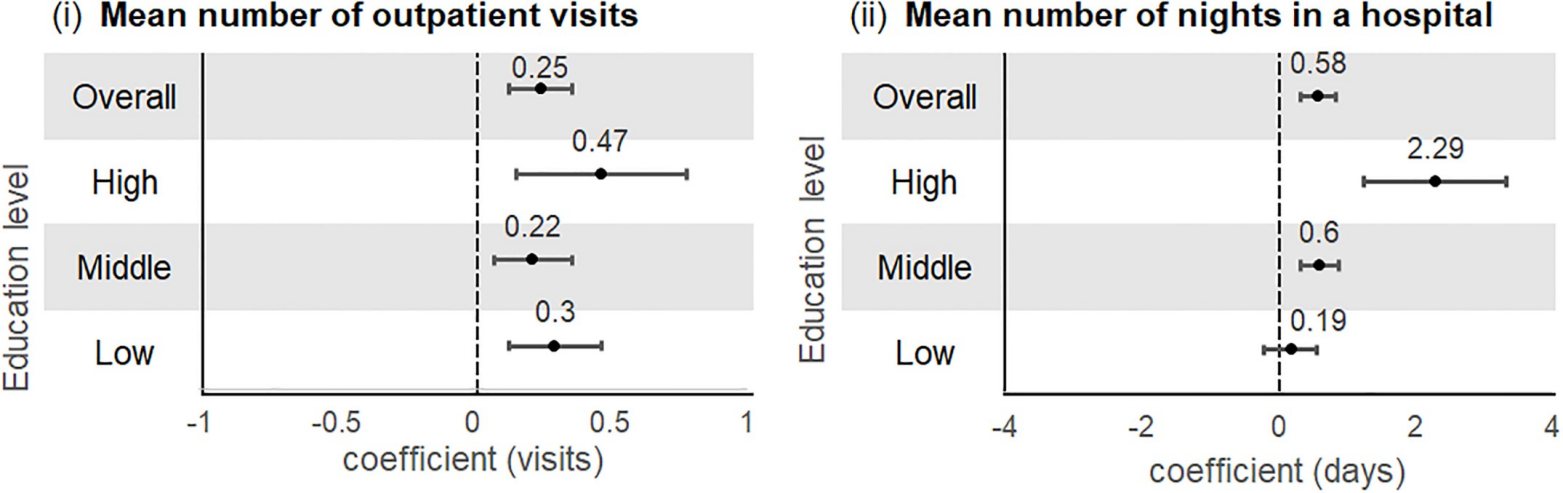
Associations between multimorbidity with mean number of (i) outpatient visits and (ii) nights in hospital by education level (Japan)^+^. ^+^Multivariable negative binomial regression models were adjusted for age, sex, marital status, employment, body mass index, physical activity, alcohol intake, and smoking.

An increasing number of NCDs was associated with a higher mean number of outpatient visits and mean number of nights in a hospital (Fig 6). There were no differences among education levels. However, there appears to be a positive trend between number of nights in the hospital with educational level, and subjects in the highest education group had a statistically significantly higher mean number of nights in a hospital (ß = 2.29, 95%CI = 1.26. to 3.33), P-value <0.001) compared to those in the middle-level education group (ß = 0.60, 95%CI = 0.32. to 0.88, P-value <0.05).

### Productivity loss

#### Australia

**Fig 7:**
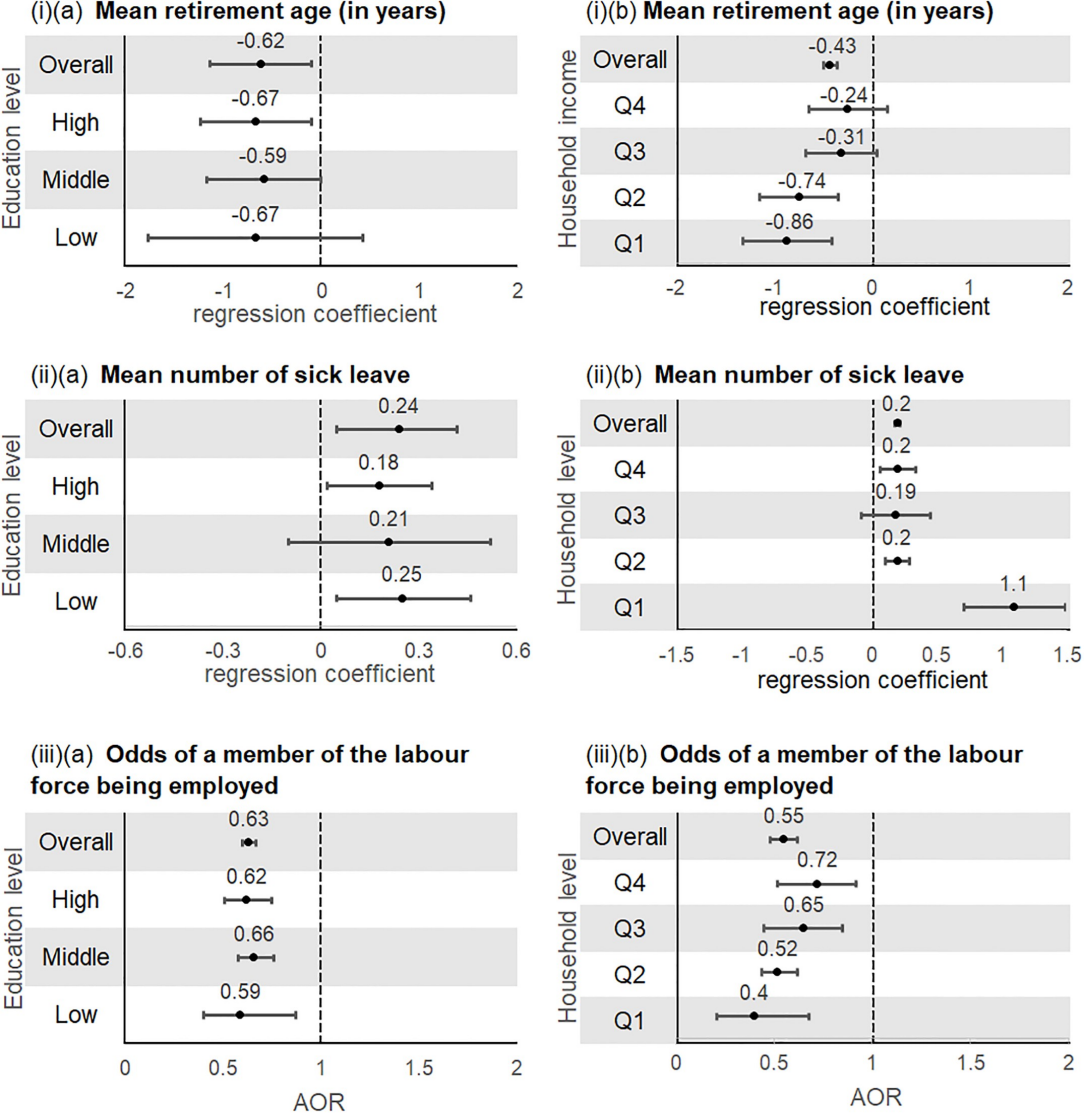
Associations between multimorbidity with (i) mean retirement age (ii) mean number of sick leave days and (iii) odds of being unemployed by (a) education level and (b) household income (Australia)^+^. ^+^Multivariable negative binomial regression models and multivariable logistic regression models were adjusted for age, sex, marital status, household income (if stratified by education)/education (if stratified by household income), geographical status, country of birth, aboriginal status, body mass index, physical activity, alcohol intake, and smoking.

An increasing number of NCDs was associated with a lower mean retirement age, greater mean number of sick leave days, and lower odds of being employed despite being in the labour force (Fig 7). There were no differences among socioeconomic groups. However there appears to be a positive trend for the outcome on mean retirement age and on the odds of being unemployed, and subjects in the lowest household income quartile have a higher mean number of sick leave days (ß = 1.1, 95%CI = 0.71 to 1.49, P-value <0.001).

#### Japan

**Fig 8:**
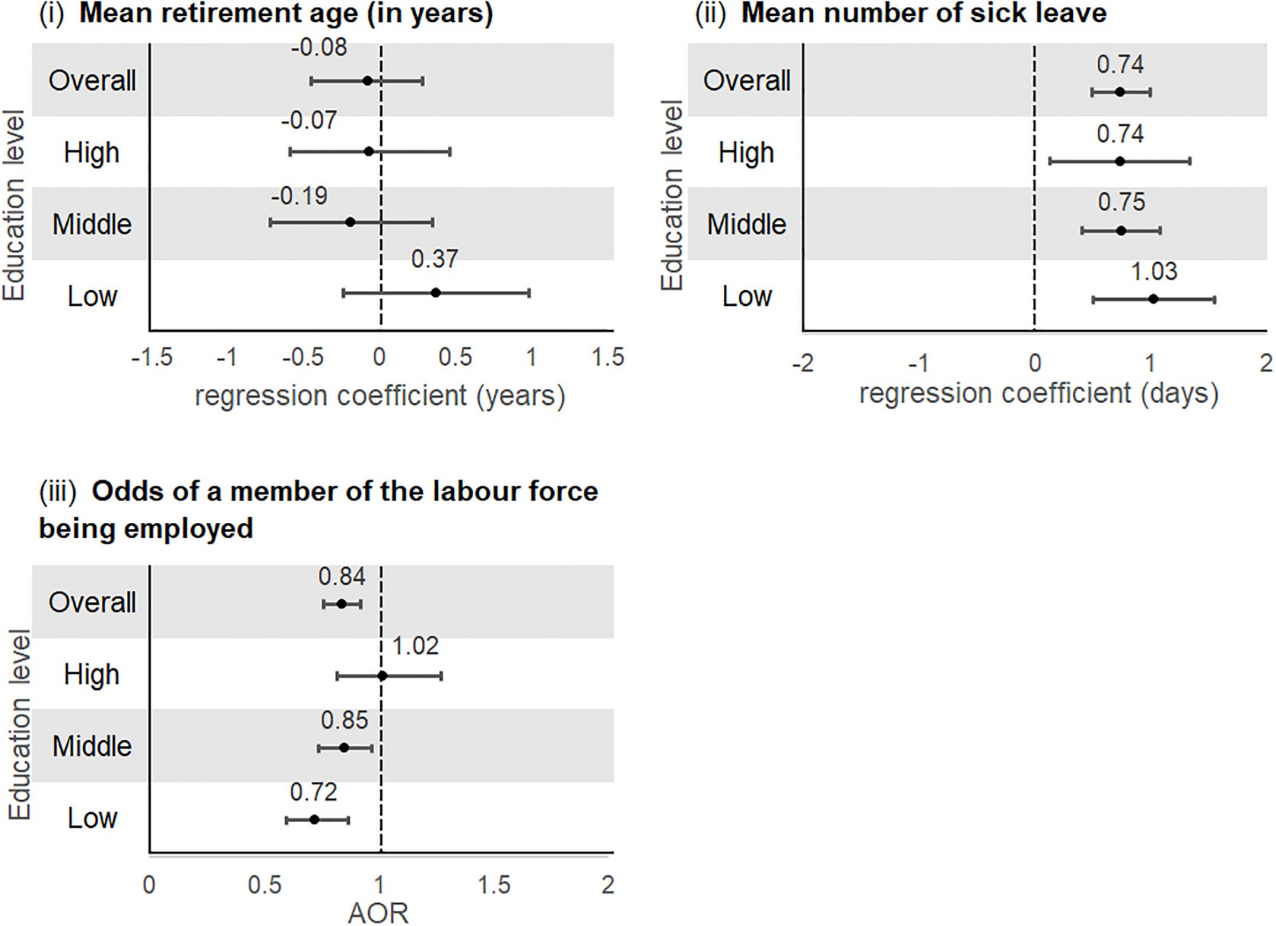
Associations between multimorbidity with (i) mean retirement age (ii) mean number of sick leave days and (iii) odds of being unemployed by education level (Japan)^+^. ^+^Multivariable negative binomial regression models and multivariable logistic regression models were adjusted for age, sex, marital status, body mass index, physical activity, alcohol intake, and smoking.

An increasing number of NCDs is associated with a greater mean number of sick leave days and lower odds of being employed despite being in the labour force (Fig 8). There is no association between more NCDs with mean retirement age. There are no differences among education levels. However, there appears to be a positive trend for the outcome on the odds of being unemployed.

## Discussion

### Principal findings

Prevalence of multimorbidity was overall 38.6% (46.0%, 36.1%, 28.9% amongst those in the lowest, middle and highest education group, respectively) in Australia, and overall 28.4% (33.9%, 24.6%, 16.6% amongst those in the lowest, middle and highest education group, respectively) in Japan. There is a higher proportion of individuals with multimorbidity in the lowest socioeconomic groups and education levels.

In both Australia and Japan, having more NCDs was associated with higher mean number of outpatient visits and number of nights in the hospital. In both Australia and Japan, more NCDs was associated with greater productivity loss, including higher mean number of sick leave days amongst the employed, and lower odds of being employed despite being in the labour force. In Australia only, having more NCDs was associated with a lower mean retirement age. Another finding is that across all levels of income and levels of education, there is an association between number of NCDs with healthcare utilisation and work productivity loss. This may appear to suggest that adults with multiple NCDs from lower socioeconomic levels could have substantial financial burden, since they have fewer resources to cope with the burden from healthcare utilisation and being unable to work.

### Comparison with literature

Our study is the first national assessment on the relationship between having more NCDs with health care utilisation and work productivity loss among adult and elderly in Australia and Japan. Our finding on the positive association between multimorbidity and healthcare utilisation is consistent with limited local studies in Australia, Japan, and other high-income countries, as are our findings that increasing numbers of NCDs are associated with more outpatient visits, more hospital admissions, and longer hospitalisations [26,33–35].

Our study findings on the adverse association between having more NCDs and productivity is consistent with the limited number of studies in the literature [14,15,36,37]. Furthermore, these limited number of studies only examined a narrow aspect of productivity loss, such as poorer work performance in employed adults [22]. Consistent with our results on multimorbidity and early retirement in Australia, a local study in Australia focused on civil servants found that older workers with chronic health conditions were less likely to work beyond 65 years of age [38]. The lack of associations between multimorbidity and early retirement in Japan may be attributed to culture and societal pressure on employers to provide employment until pension age, and stigma of early retirement and unemployment [39,40]. Another plausible explanation is that unlike Australia, subjects in Japan may delay retirement as they have an incentive scheme to work beyond retirement age and they do not have asset and income limits to pension payouts [41–43].

Third, another finding is that there is an association between number of NCDs with healthcare utilisation and work productivity loss, across all levels of income and levels of education. This finding may appear to suggest that individuals with the least resources to cope with the burden from healthcare utilisation and work productivity loss, may face more substantial financial hardships from having multiple NCDs. A study in South Korea showed that having catastrophic health expenditures is associated with having an individual with a chronic disease in the household, being of a lower socioeconomic status, and/or having an elderly person in the household [44]. A study that examined catastrophic health payments in 59 countries found that risk factors include the over-reliance of health systems on out-of-pocket payments, low capacity of households to pay, and the general lack of health insurance and financial risk protection [45]. Having catastrophic health expenditures is an important issue for healthcare systems as it is associated with severely reduced quality of life and it drives impoverishment [46–50].

### Study limitations

Self-reported medical history may be poorly correlated to medical status, and likely more so in less educated, poor, and rural populations [51]. Outpatient visits were not specific for NCDs and might include visits for other unrelated conditions [27,52]. Hospitalisations were studied as the number of nights in the hospital, and the study did not examine the number of episodes of hospitalisation, or the severity and nature of each hospitalisation [27,52]. Productivity loss in terms of individuals in the labour force being unemployed due to other non-chronic conditions like acute and infectious illnesses, or physical injury unrelated to NCDs [27,52]. However, it is much less likely for individuals in the labour force to face unemployment due to acute and infectious conditions. Another study limitation was that work performance or on-the-job productivity was not examined, as this was not assessed by the surveys. Self-reported number of sick leave days may be subject to recall error. Actual retirement age may be younger than what respondents self-report (respondents may self-report a higher age) due to social desirability bias and not accurately reporting being unemployed or having involuntary early retirement [39,40].

A direct comparison between the results from HILDA and JSTAR was limited by the differences in the surveys, in terms of the exact wording of questions and number of questions for each outcome measure. However, our findings were still able to show how outcomes can be similar across high-income countries.

This study was based on a limited number of NCDs, so further work could examine more conditions, like the large-scale Scotland study with 40 NCDs [53]. The cross-sectional design does not allow for causal interpretations, and further studies that use prospective cohort designs are needed to examine how multimorbidity causes healthcare utilisation and productivity loss over an individual’s life course [3].

### Policy, clinical, and research implications

Our study presents evidence that a higher proportion of individuals with less education experience more NCDs. In addition, it appears that across all levels of income and levels of education, there is an association between number of NCDs with healthcare utilisation and work productivity loss. This may suggest that those from lower socioeconomic levels could have more financial burden, because they tend to have fewer resources to cope with greater healthcare utilisation and forced to exit the labour force. Policymakers should consider health financing strategies, such as the removal of user charges or subsidising for the poorer population [11,54]. Flexible payment plans that allow instalments, subsidised premiums, and removal of co-payments, are measurements that can reduce the catastrophic financial burden by the poor [48]. The substantial burden of more healthcare utilisation from multimorbidity in Australia and Japan, need urgent addressing. Health systems, from a planning, delivery and evaluation perspective, need to shift from single-disease models to a paradigm that account for the complexity of multimorbidity [3,55].

Clinical guidelines for patients with multiple NCDs could be refined to consolidateand coordinate the management of multiple NCDs, and have patient-centred approaches (e.g. reflecting patients’ preference in treatments and medications) which minimise the impact of multimorbidity on high healthcare utilisation [55–57].

Our findings showing that having more NCDs is associated with substantial productivity loss highlighted the implications and costs of multimorbidity goes beyond health system but also on the individual and household finance, and wider society [14,15,25,58]. Policies need specific aims on curbing involuntary early retirement, absence from work, and not being employed despite being in the labour force and actively seeking employment [14,18,25,58,59].

Employers and governments should be aware that having more NCDs affects work force productivity and implement prevention programmes to reduce impact of chronic conditions on the workforce. For instance, employers could implement health programmes that promote healthier lifestyles, such as more balanced diets, increased physical activity, and workplace environmental modifications that reduce sedentary behaviour [60–62]. Policies are needed to motivate companies and relevant stakeholders to implement flexible work schedules for workers who need time off for treatment and therapies [63], in order to minimise unnecessary forced unemployment. Strategies to mitigate the adverse effect of multimorbidity on productivity should be considered as investment rather than costs.

Clinicians should consider the impact on patients’ employment (impact on sickness absence or needing to leave employed work) Clinicians should take into account the challenges of treatment, such as potential side effects from medications which affect cognitive function and physical agility, and time-consuming treatments which require a substantial time away from work, like kidney dialysis or chemotherapy.

As this is one of first studies that used nationally representative data to examine work productivity loss, and there is still a major dearth in the literature on the impact of multimorbidity on productivity loss in both HICs and MICs on nationally representative samples. Hence, future chronic disease surveys need to include questions on frequency and duration of sickness absence, not being employed due to NCDs, and early retirement. There should also be questions pertaining to work performance or on-the-job productivity. While this paper examined how having more NCDs was linked to unemployment and early retirement, further investigation can be conducted on quantifying the loss of income from being unemployed and retiring early for persons with different numbers and clusters of NCDs. Additionally, building on our study, further studies could investigate the financial impoverishment from healthcare utilisation and productivity loss faced by persons with multiple NCDs in the lower socioeconomic groups. Sex differences, such as differences in retirement age or being unemployed in males versus females would also be interesting to investigate in future work.

Existing studies on work productivity loss have been primarily on the impact of self-perceived ill health or single NCDs like hypertension, diabetes, or mental illness [23,24]. Additionally, majority of the existing literature focuses on the working population (i.e. employees), with a dearth in studies on how having more NCDs impacts the involuntary exit from the labour force from unemployment and early retirement [21,64]. Hence, future studies can build on this paper by looking at the impact of NCD combinations, as well as nationally representative data from other countries. Future research work could also examine additional aspects of healthcare utilisation, such as pharmaceuticals, laboratory testing, and medical equipment.

## Conclusion

Having more NCDs poses significant economic burden to the health system and wider society in Australia and Japan, and the impact appears to occur across all socioeconomic groups. Decisive action is critical for improving universal health coverage and improve financial protection, especially for lower income groups who are more likely to have multiple NCDs. These individuals incur both high direct and indirect costs, which lead to a greater risk of impoverishment.

## Supporting information

S1 Table Survey questions for productivity loss. (DOCX)

S2 Table Multivariable regression models used in study. (DOCX)

S1 Fig Sample flowchart (Australia). (TIF)

S2 Fig Sample flowchart (Japan). (TIF)

S3 Fig Prevalence of individual non-communicable diseases (Australia). (TIF)

S4 Fig Prevalence of individual non-communicable diseases (Japan). (TIF)

## Abbreviations

HILDA: Household, Income, and Labour Dynamics Australia
JSTAR: Japanese Study of Ageing and Retirement
NCDs: Non-communicable diseases
