# Supplementary material for: Implications of multimorbidity on healthcare utilisation and work productivity by socioeconomic groups: Cross-sectional analyses of Australia and Japan
Source: PLoS One. 2020 Apr 28;15(4):e0232281. doi: 10.1371/journal.pone.0232281 (PMC7188213; doi:10.1371/journal.pone.0232281)
Supplement: S1 Table — (DOCX) [file pone.0232281.s001.docx]

**Appendix Table 1**. Survey questions for productivity loss

|  | **Australia**  **(HILDA survey)** | **Japan**  **(JSTAR survey)** |
| --- | --- | --- |
| Mean retirement age (years) | Subjects self-reported being retired by answering affirmatively to: “Have you retired from the workforce?”.  Retired subjects responded to the subsequent question: “In which year did you retire?”. | Subjects self-reported being retired by answering affirmatively to: “Did you retire from the job due to age?”.  Retired subjects responded to the subsequent question “When did you reach retirement age (please provide month and year)?”. |
| Mean number of days of sick leave in the past 12 months | Subjects were asked: “How many weeks or days did you spend on paid sick leave in the past 12 months?”. | Subjects were asked: “At the job, did you take any days off due to sickness during the past 12-month period? If you did, how many days did you take?”. |
| Odds of being unemployed despite being in the labour force (Yes/No) | Respondents who answered affirmatively to: “being employed or seeking work the past 4 weeks”, were subsequently asked for the reason they have not been able to work or successfully look for work the past 4 weeks.  Respondents who selected the option “illness, injury, or disability” were categorised as being in the labour force but were not employed due to illness. | Respondents were asked: “Are you currently working? Please answer yes if you work at all, even if you receive no pay. If you are temporarily not working due to illness, please select 2 as the answer to this question.”  The options were (1) Yes; (2) Temporarily not working; (3) No; (4) Do not know; (5) Refused to answer.  Respondents who selected option (2) were categorised as being in the labour force but were not employed possibly due to illness. |

HILDA= Household, Income, and Labour Dynamics Australia; JSTAR= Japanese Study of Ageing and Retirement
