# Supplementary material for: Implications of multimorbidity on healthcare utilisation and work productivity by socioeconomic groups: Cross-sectional analyses of Australia and Japan
Source: PLoS One. 2020 Apr 28;15(4):e0232281. doi: 10.1371/journal.pone.0232281 (PMC7188213; doi:10.1371/journal.pone.0232281)
Supplement: S2 Table — (DOCX) [file pone.0232281.s002.docx]

**Appendix Table 2.** Multivariable regression models used in study

| **Study outcome** | **Multivariable regression model** |
| --- | --- |
| **Healthcare utilisation**  **in the past 12 months** |  |
| Mean number of outpatient visits | Negative binomial |
| Mean number of nights in a hospital | Negative binomial |
|  |  |
| **Productivity loss** |  |
| Mean retirement age (years) | Linear |
| Mean number of sick leave days in the past 12 months | Negative binomial |
| Odds of being unemployed despite being in the labour force (Yes/No) | Logistic |
