# Supplementary figures and images for: Implications of multimorbidity on healthcare utilisation and work productivity by socioeconomic groups: Cross-sectional analyses of Australia and Japan
Source: PLoS One. 2020 Apr 28;15(4):e0232281. doi: 10.1371/journal.pone.0232281 (PMC7188213; doi:10.1371/journal.pone.0232281)

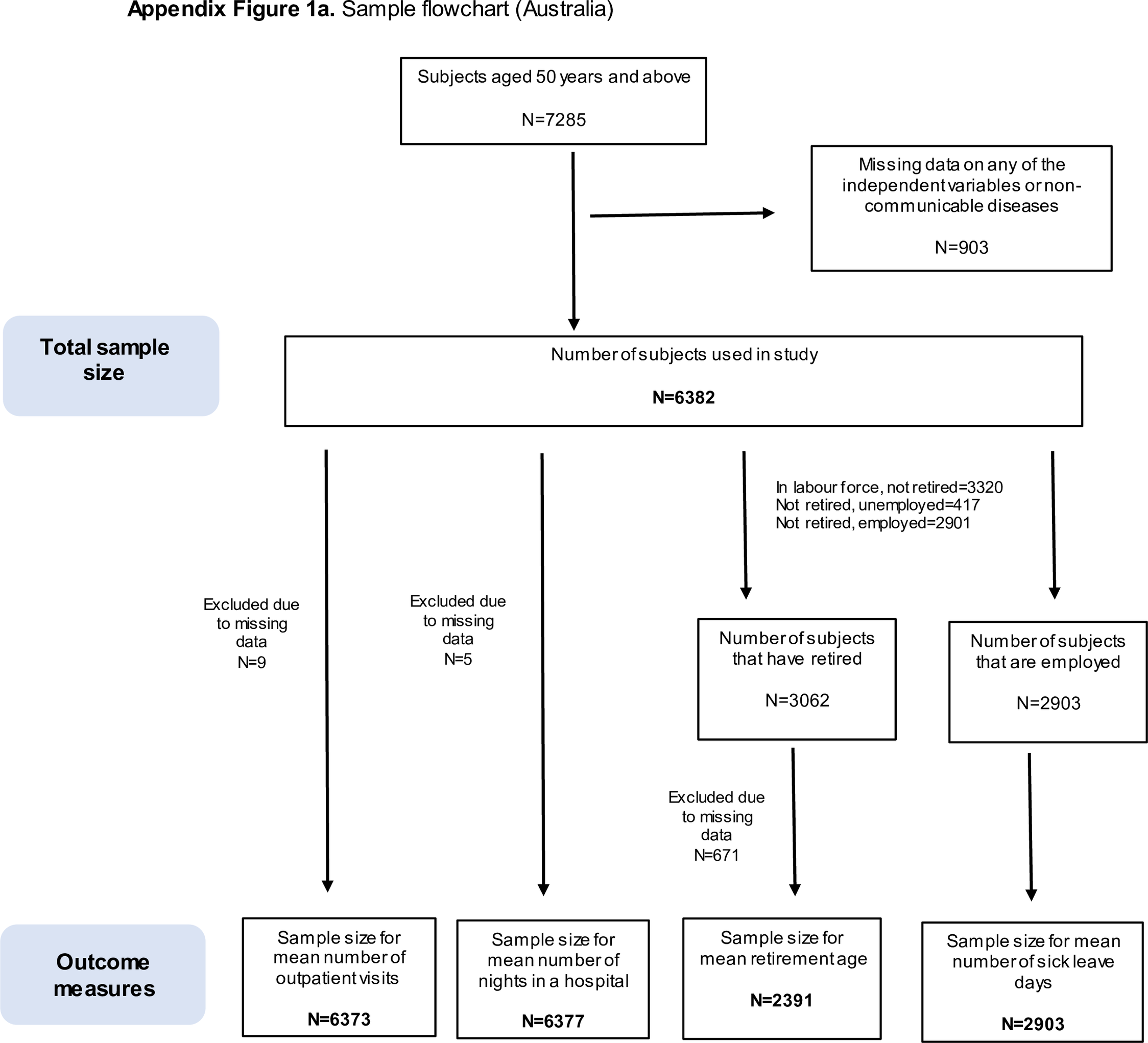

Supplement: S1 Fig — (TIF) [file pone.0232281.s003.tif]

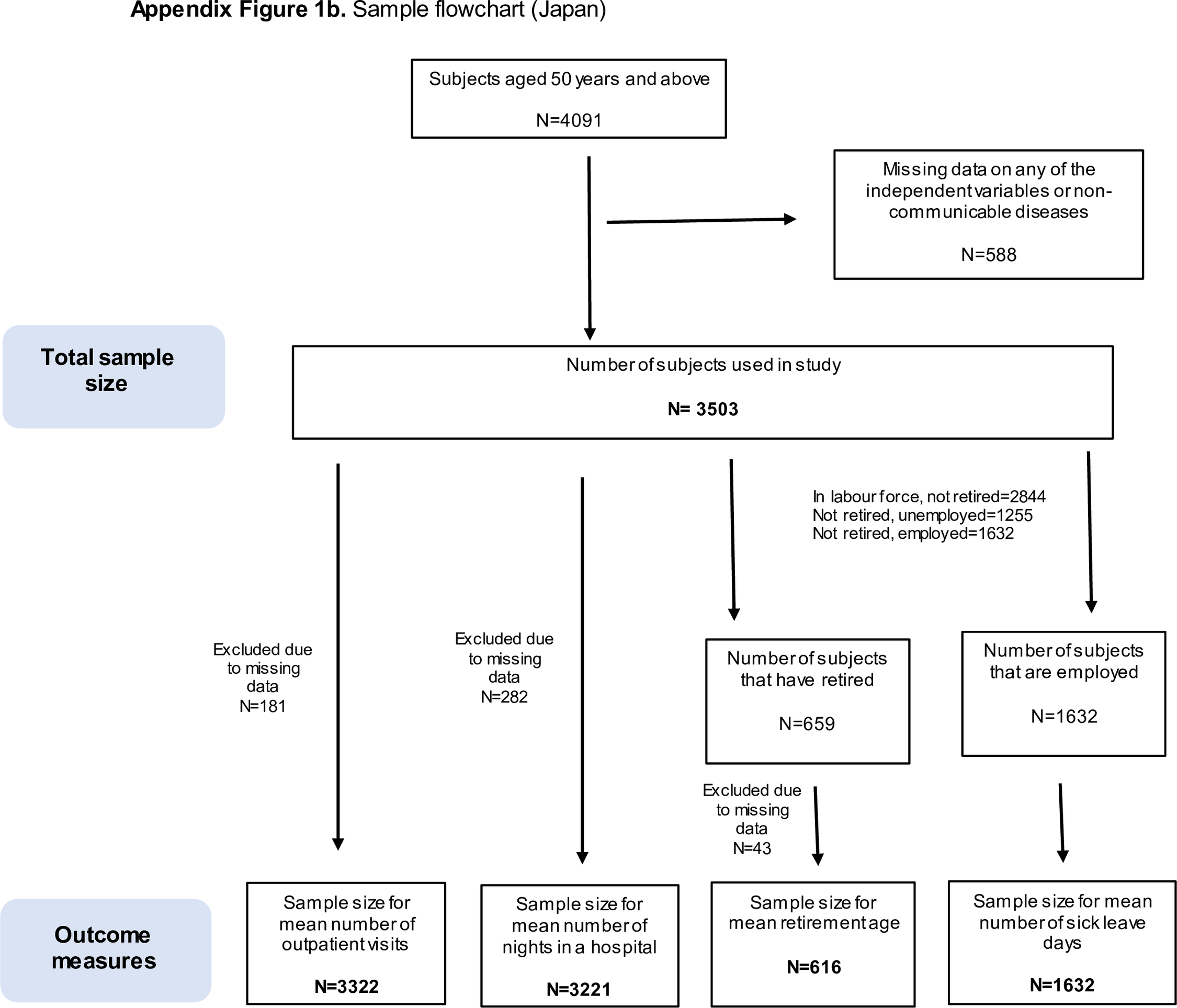

Supplement: S2 Fig — (TIF) [file pone.0232281.s004.tif]

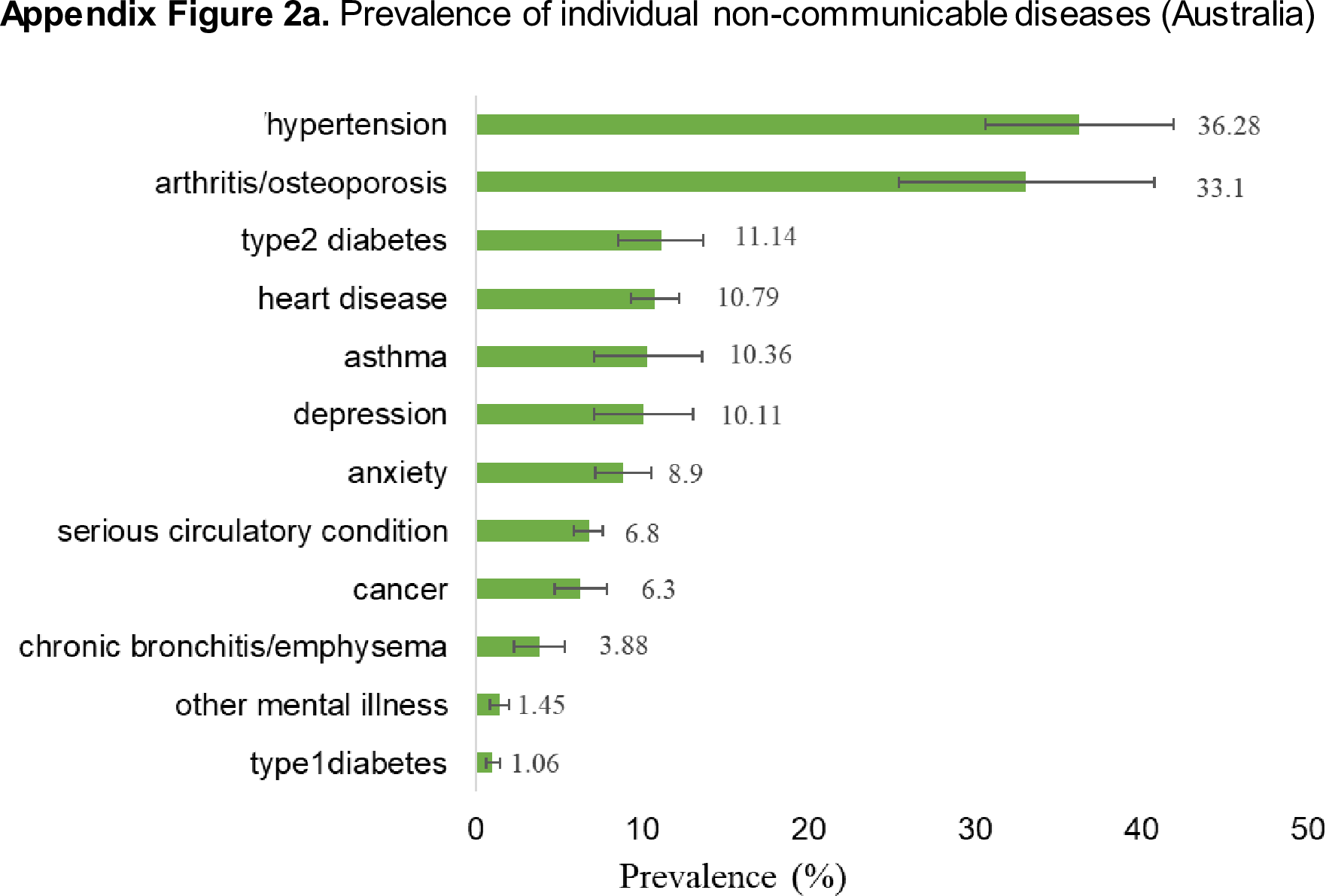

Supplement: S3 Fig — (TIF) [file pone.0232281.s005.tif]

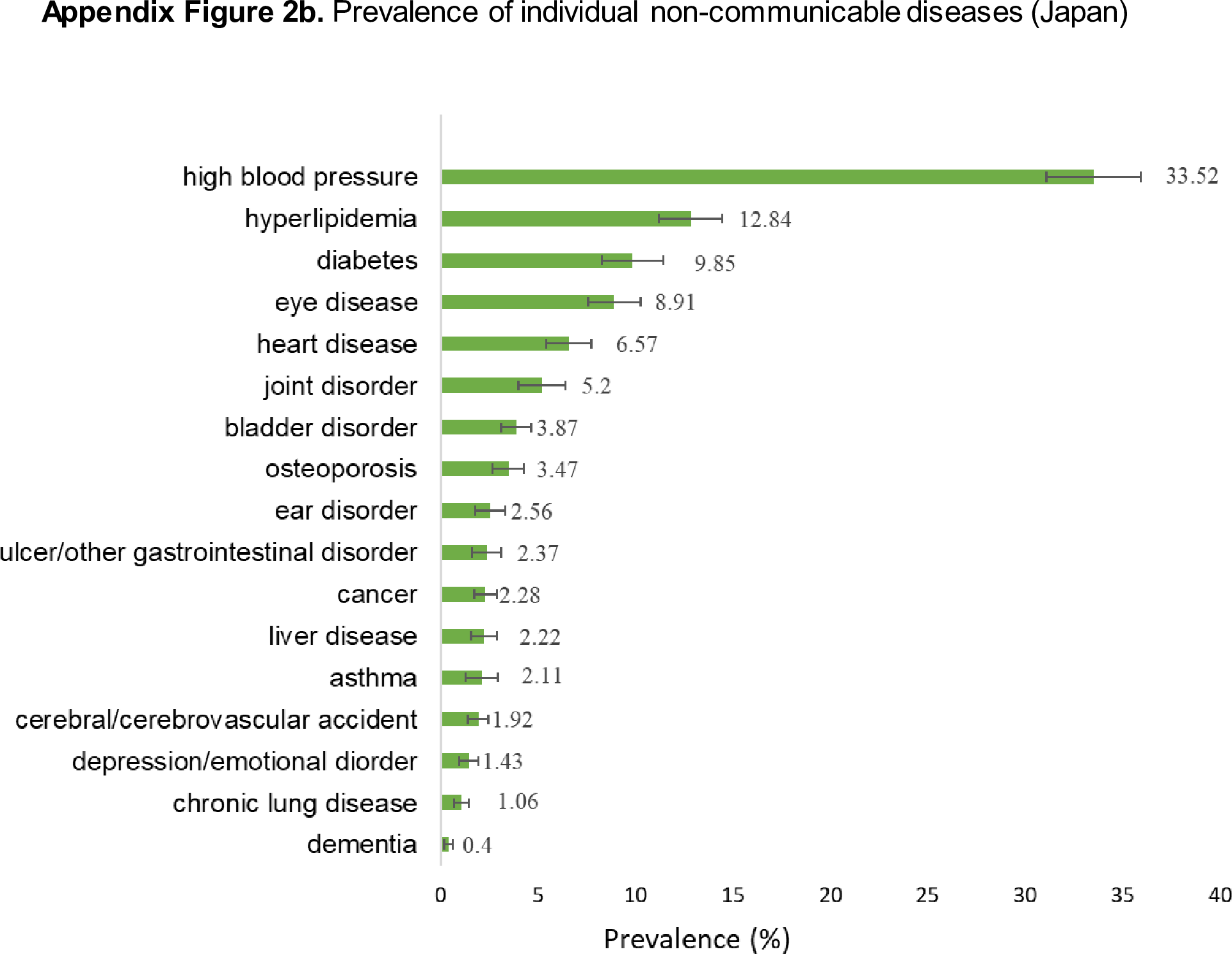

Supplement: S4 Fig — (TIF) [file pone.0232281.s006.tif]
